# Molecular Quantification and Genetic Diversity of Toxigenic Fusarium Species in Northern Europe as Compared to Those in Southern Europe

**DOI:** 10.3390/microorganisms1010162

**Published:** 2013-12-03

**Authors:** Tapani Yli-Mattila, Sari Rämö, Veli Hietaniemi, Taha Hussien, Ana Liza Carlobos-Lopez, Christian Joseph R. Cumagun

**Affiliations:** 1 Molecular Plant Biology, Department of Biochemistry, University of Turku, FI-20014 Turku, Finland; 2 MTT Agrifood Research Finland, FI-31600 Jokioinen, Finland; E-Mails: sari.ramo@mtt.fi (S.R.); veli.hietaniemi@mtt.fi (V.H.); 3 Mycotoxins Lab, Department of Food Toxicology and Contaminant, National Research Center, Cairo 12311, Egypt; E-Mail: taha_bio@yahoo.com; 4 Crop Protection Cluster, College of Agriculture, University of the Philippines Los Baños, Laguna 4030, Philippines; E-Mails: ana_lizalopez@yahoo.com (A.L.C.-L.); cumagun@daad-alumni.de (C.J.R.C.)

**Keywords:** *Fusarium*, mycotoxins, diversity, Europe, qPCR

## Abstract

*Fusarium* species produce important mycotoxins, such as deoxynivalenol (DON), nivalenol (NIV) and T-2/HT-2-toxins in cereals. The highest DON and T-2/HT-2 toxin levels in northern Europe have been found in oats. About 12%–24% of Finnish oat samples in 2012 contained >1.75 mg·kg^−1^ of DON, which belongs to type B trichothecenes. *Fusarium graminearum* is the most important DON producer in northern Europe and Asia and it has been displacing the closely related *F. culmorum* in northern Europe. The 3ADON chemotype of *F. graminearum* is dominant in most northern areas, while the 15ADON chemotype of *F. graminearum* is predominating in Central and southern Europe. We suggest that the northern population of *F. graminearum* may be more specialized to oats than the southern population. Only low levels of *F. culmorum* DNA were found in a few oat samples and no correlation was found between *F. culmorum* DNA and DON levels. DNA levels of *F. graminearum* were in all cases in agreement with DON levels in 2011 and 2012, when DON was measured by gas chromatography-mass spectrometry (GC-MS). When the *RIDA*^®^ *QUICK* SCAN kit results (DON) were compared to DNA levels of *F. graminearum*, the variation was much higher. The homogenization of the oats flour by grinding oats with 1 mm sieve seems to be connected to this variation. There was a significant correlation between the combined T-2 and HT-2 and the combined DNA levels of *F. langsethiae* and *F. sporotrichioides* in Finland in 2010–2012.

## 1. Introduction

### 1.1. Type B Trichothecene Producers and Their Chemotypes

All species of *F. graminearum* species complex (FGSC) produce type B trichothecene mycotoxins, such as DON (deoxynivalenol) and NIV (nivalenol). Three chemotypes have been recognized among isolates: producers of DON and 3ADON (chemotype 3ADON), producers of DON and 15ADON (chemotype 15ADON), and producers of NIV and 4ANIV (chemotype NIV). Many species contain isolates of all chemotypes, whereas others, such as *F. meridionale*, contain only NIV chemotype [1]. *F. graminearum* sensu stricto has been the dominant FGSC species in all investigations dealing with Europe in Hungary, Serbia-Montenegro and Austria [2,3], Finland, Russia [4] and Germany [5,6]. *F. asiaticum* together with *F. ussurianum*, *F. vorosii* and *F. nepalense* form the Asian subclade, which is endemic in Asia. The species of this subclade cause Fusarium head blight of cereals in large areas including China, Japan, Korea, Nepal and the Russian Far East [4,7].

In addition to *F. graminearum*, *F. culmorum* produces type B trichothecenes, but only two chemotypes are known in this species: NIV and 3ADON [5,8], except those from Turkey [9], where the 15ADON chemotype of *F. culmorum* has been found. *F. cerealis* is strictly a NIV chemotype [5,6,10].

**Figure 1:**
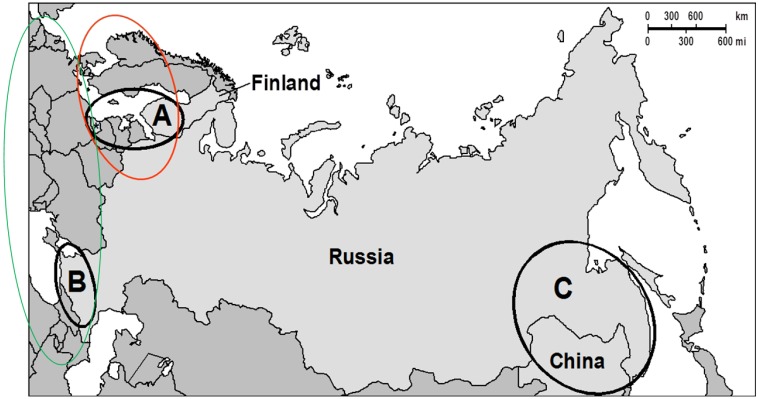
In the population of northern Europe (*red circle*), 3ADON molecular chemotype of *F. graminearum* is dominant, while 15ADON molecular chemotype is more common in the population of southern and central Europe (*green circle*) including Turkey and northwestern Iran. *F. ussurianum* (3ADON genotype) and *F. vorosii* (15ADON genotype) were also found in Russian Far East. *A*, *B* and *C* represent the three main areas, where *F. graminearum* isolates were collected by Yli-Mattila *et al.* [4] for chemotype analyses.

The 3ADON chemotype of *F. graminearum* is predominating in most northern areas (Figure 1), such as Finland, Norway, Sweden, North-western Russia, Russian Far East, northern Japan and some parts of Canada [6,9,11]. 3ADON isolates of *F. graminearum* and *F. culmorum* inhibit the growth of wheat seedlings more strongly and cause more necrotic lesions than 15ADON isolates. This is in agreement with higher DON production of 3ADON isolates [1,6]. The 3ADON chemotype is also growing more quickly and it produces more conidia than the 15ADON chemotype, which might explain, why the 3ADON chemotype is spreading in Russian Far East and North America.

### 1.2. Historical Background

In Denmark highest DON levels were found in wheat in 1997–2000 and 2004 [12]. In Sweden the quality of the oat yield was exceptionally poor in 2011 and an increase in mycotoxin levels was found in oats, when harvesting was delayed due to the rains. The highest DON levels were found in Finland in 2000, 2005, 2007, 2011 and 2012 [13,14]. In 2012, the exceptionally high DON levels in oats could be attributed to the exceptionally high precipitation and humidity levels in Finland. In 2000 high NIV levels (200–3700 ppb) were also detected in barley in Finland and in North-western Russia [15]. High NIV levels were also found in South-western Finland in 2002 both due to natural high *F. poae* levels and in barley plots, in which *F. poae* was induced by artificial inoculation by different *Fusarium* species [16].

In central and southern Europe, the 15ADON chemotype has been predominating (Figure 1), while the 3ADON chemotype has been rarely found in Germany [6,7,17], England [18], Poland [10], Hungary [3], Luxembourg [19] and Italy [20]. In western Europe the NIV chemotype has also been relatively common.

In Denmark, *Fusarium* species and their chemotype composition were analysed from historical samples by using qPCR analyses [12,21]. According to these results, the 3ADON chemotype of *F. culmorum* dominated in 1957–1960 and in 1977–2000 in wheat. It was only in 1997–2000 that the 15ADON chemotype was found at the same time as *F. graminearum* started to appear. During the years 1965–1968, *F. graminearum* was more common than *F. culmorum* in wheat, but at that time *F. graminearum* belonged to 3ADON chemotype, because no 15ADON chemotype was found. After 2003 the 15ADON chemotype of *F. graminearum* has been the most common chemotype in wheat in Denmark. In barley the 3ADON chemotype of *F. culmorum* has been predominating all the time, but in 1965–1968 the 3ADON chemotype of *F. graminearum* was also found and after 2005 the 15ADON chemotype of *F. graminearum* was also found in barley. Oats are the only cereal in which the 3ADON chemotype of *F. graminearum* remains dominant in Denmark, although the 3ADON chemotype of *F. culmorum* and the 15ADON chemotype of *F. graminearum* are also present.

Recently, *F. graminearum* has been spreading northward in Europe (e.g., [6,10,12,22,23]) displacing the closely related *F. culmorum.* This shift may be due to changing agricultural practices, climate change and increased maize cropping. In Finland, *F. graminearum* has already been reported in cereals in 1932 [24] and in the 1960s [25], and it has been present since then [26,27,28,29,30], while in North-western Russia *F. graminearum* was found only a few years ago [4,6]; thus the north-western Russian *F. graminearum* population may have originated from Finland. *F. graminearum* is also present in Norway, Sweden and Estonia, but not in Lithuania [31,32]. According to Aamot *et al.* [33] the 15ADON chemotype of *F. graminearum* has also been found in Norway from recently collected isolates like in the case Denmark ten years earlier. The 15ADON population in Norway may have originated from central Europe. The 15ADON chemotype of *F. graminearum* is the most common chemotype in Turkey [34] and northwestern Iran, while the NIV chemotype is dominant in northern Iran [35].

### 1.3. Type A Trichothecene Producers

Species related to *F. sporotrichioides* produce type A trichothecenes, such as T-2 and HT-2 toxins. New T-2/HT-2 toxin-producing *Fusarium* species (*F. langsethiae* and *F. sibiricum*) have been recently found in northern Europe and Asia [36] and a similar isolate was also found in Iran [37]. *F. langsethiae* is mainly distributed in Europe, while *F. sibiricum* is mainly distributed in Siberia and Russian Far East [36]. *F. poae* is an intermediate between type A and B producers. It can produce *in vitro* DAS, which is a type A trichothecene, but it is also the main NIV-producer in northern Europe [38,39,40] and northern Japan [41]. NIV, unlike DON, occurs more frequently after dry and warm growing seasons [42].

### 1.4. Genetic Basis of Chemotypes

A big cluster of trichothecene genes is required for trichothecene synthesis. *TRI7* and *TRI13* genes are involved in NIV and T-2 toxin synthesis, while *TRI16* gene is necessary for T-2 toxin synthesis. DON is produced in *F. graminearum* instead of NIV, if *TRI7* and *TRI13* genes are nonfunctional [43,44]. Differences in activity of the esterase encoded by *TRI8*, which are due to differences in the DNA sequences of the *TRI8* gene, determine whether 3ADON or 15ADON is produced by the isolates [45]. DON often accumulates in cereal grains, presumably because host plant or fungal esterases (deacetylases) remove the acetyl units from 3ADON or 15ADON.

### 1.5. The Aim of the Work

In the present work *Fusarium* mycotoxins and the fungal DNA levels and the correlation between them were monitored in Finnish oats samples during the years 2010, 2011 and 2012. Based on the results of the present work and those of previous investigations it is suggested that the *F. graminearum* isolates in Europe can be divided into two main populations, which may have specialized to different host plants. In addition, the effect of using 1 mm sieve during oat milling on DNA levels was investigated.

## 2. Experimental Section

### 2.1. Fusarium Isolates

The single spore isolates S55, G243 and C192 used for standards in qPCR were from Finland and Russia.

### 2.2. Mycotoxin Analyses

Ten oat grain samples of the year 2010, 15 samples of the year 2011 and 40 samples of the year 2012 were analyzed for DON, 3ADON, 15ADON, T-2 and HT-2 toxins at MTT Agrifood Research Finland (Jokioinen, Finland; Tables S1–S5). In addition in 20 oat samples of the year 2012 the DON level was estimated by using the *RIDA*^®^ *QUICK* SCAN (R-Biopharm AG, Darmstadt, Germany). In MTT the trichothecenes were extracted and analyzed with GC-MS as described by Hietaniemi *et al.* [46] and Yli-Mattila *et al.* [32]. In MTT the grain flour was ground by a mill and passed through a 1 mm sieve in 2012 (2 mm sieve earlier) in order to homogenize it prior to mycotoxin and DNA measurements and to achieve better repeatability. The milled flour of the MTT samples was also used for DNA measurements, while for the samples from the food company the flour was only ground by mill prior to toxin measurement and it was not homogenized by a 1 mm sieve. 

### 2.3. DNA Extraction

*F. graminearum*, *F. sporotrichioides* and *F. culmorum* isolates used for standards were cultured on potato dextrose agar (PDA; Scharlau Chemie S.A., Barcelona, Spain) at 25 °C. DNA was extracted with the GenElute™ Plant Genomic DNA Kit (Sigma-Aldrich, St. Louis, MO, USA) as described by Yli-Mattila *et al.* [38].

### 2.4. Quantitative PCR

Oats flour ground in MTT was used for DNA extraction, while the food company oats grains were ground at the University of Turku (Turku, Finland) without sieving. DNA was extracted from ground grain samples by using GenElute™ Plant Genomic DNA kit of Sigma as described by [14]. Grain samples containing different levels of DON and/or T-2 and HT-2 toxins (Tables S1–S5) were selected for the correlation analysis. Total DNA of standards and grain samples was quantified by using a fluorescence-based Qubit fluorometer (Invitrogen, Carlsbad, CA, USA) as described by Yli-Mattila *et al.* [32].

The TMFg12 primers and probe have been designed for the *F. graminearum* specific RAPD-PCR product [37]. The TMLAN primers and probe for *F. langsethiae/F. sporotrichioides* have been designed by Halstensen *et al.* [47] and the culmorumMGB primers and probe for *F. culmorum* by Waalwijk *et al.* [48]. A Bio-Rad IQTM5 Real-Time PCR Detection System (Bio-Rad, Hercules, CA, USA) was used for running qPCR samples. DNA level of *Fusarium* species in grain samples was counted per total DNA quantified by Qubit fluorometer. All grain samples from 2010, 15 samples from 2011 and 38 samples from 2012 were analyzed by using *TMFg12* primers and probe, while only 18 samples from 2012, 18 samples from 2011 and 10 samples from 2010 with high T-2/HT-2 levels were analyzed by using *TMLAN* primers and probe.

*R*^2^ (coefficient of determination), regression slope and *p* (significance of regression slope) were calculated using the program SigmaPlot version 12.0 (SPSS Inc., Chicago, IL, USA). The original DNA and toxin concentrations were transformed to logarithmic values [1 + lg(*x*)] in order to obtain a more normal distribution for the values of toxin and DNA concentrations. 

## 3. Results and Discussion

### 3.1. The Correlation between Mycotoxin and *Fusarium* DNA Levels in 2010–2012

**Figure 2:**
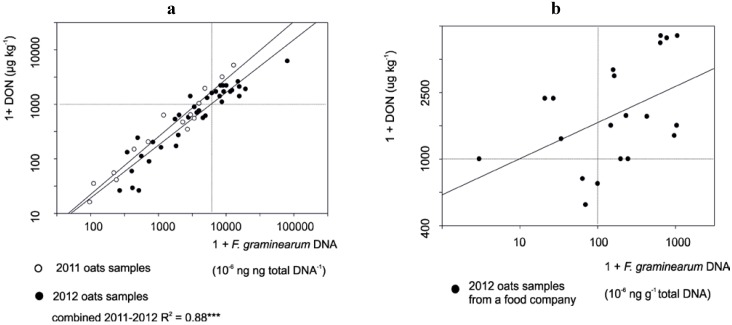
Correlation between log *F. graminearum* DNA and deoxynivalenol (DON) in Finnish oat grain samples in 2011–2012. In (**a**) the mycotoxin levels were measured at MTT by using GC-MS [46]; while in (**b**) DON level was estimated by using the *RIDA*^®^ *QUICK* SCAN. Regression slopes and *R*^2^ (coefficient of determination) values are shown. *** means highly significant (*p* < 0.001).

**Figure 3:**
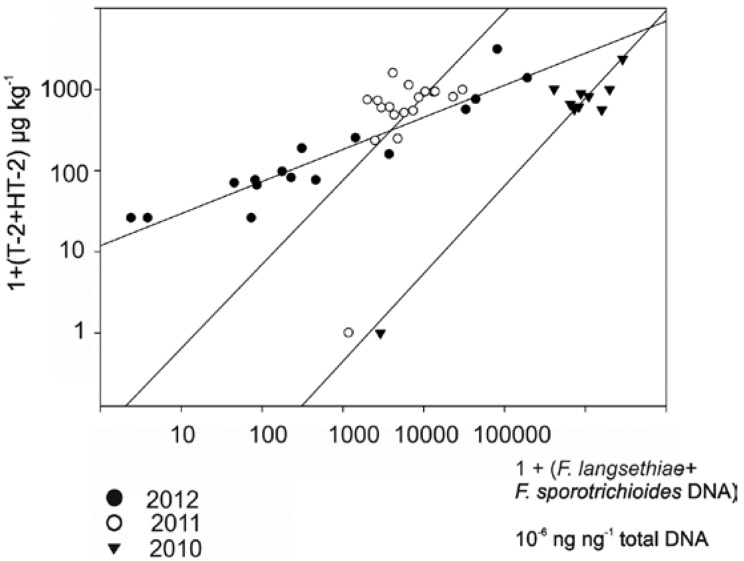
Correlation between *TMLAN* DNA and HT-2/T-2 levels in oats in 2010, 2011 and 2012. *R*^2^ = 0.94 *** (2010), 0.30 * (2011), 0.89 *** (2012). Regression slopes are shown. *** means highly significant (*p* < 0.001) and * means significant (*p* < 0.05).

The highest levels of deoxynivalenol (DON) were found in Finnish oats in 2012, when about 10%–25% of oats samples contained >1.75 mg·kg^−1^ of DON [13]. In the samples from MTT the DON levels varied between <25 and 10000 ppb (Figure 2a) and the T-2/HT-2 toxin levels between <25 and 3000 μg·kg^−1^ (Figure 3). In the oats samples from a food company the DON levels ranged between 530 and >5500 μg·kg^−1^ (Figure 2b). The coefficient of determination (*R*^2^) between *F. graminearum* DNA and DON levels was 0.95 *** (*p* < 0.001) from 15 oats samples from 2011 and 0.87 *** from 40 oats from 2012 obtained from MTT (Figure 2a), while the *R*^2^ value from 20 oats samples from a food company was only 0.22 * (*p* < 0.05, Figure 2b).

The difference in correlation may be at least partially caused by different milling methods, which is supported by our previous results. The correlation between DON and *F. graminearum* levels was lower, when DNA was extracted from oats flour, which was ground by a coffee mill without a sieve [14] as compared to the present paper, in which both DNA and DON were extracted from the oats flour obtained by a mill having a 1 mm sieve for homogenization. According to Rauvola *et al.* [49] and Rauvola [50], the correlation between *RIDA*^®^ *QUICK* SCAN and GC-MS results was good, when, in both cases, DON levels were measured from oats flour ground with a 1 mm sieve. Finnish food companies, however, have been using *RIDA*^®^ *QUICK* SCAN for oats samples ground without the 1 mm sieve, which might have caused variation in results.

According to Rauvola [50] *RIDA*^®^ *QUICK* SCAN is often giving in oats DON levels, which are above the EU limit (1.75 mg·kg^−1^), while according to GC-MS results, the DON levels are only close (1.4–1.7 mg·kg^−1^) to the EU limit, which might at least partially explain, why according to food companies *ca.* 25% of oats samples from 2012 in Finland contained too much DON for human food [51], while according to the MTT [13] only *ca.* 10% of oats samples exceeded the limit. Lower DON values with *RIDA*^®^ *QUICK* SCAN were also obtained in wheat and barley [50], especially close to the EU limit (1.25 mg·kg^−1^) [52]. It should also be taken into consideration that there may be variation even in GC-MS results between different measurements in different laboratories. These may be e.g., due to differences in sample preparation and sample selection. For consumers the lower DON values guarantee that that the DON values are below the EU limit, but for farmers they mean that the yield may be rejected due to false positive DON values. Thus, both *RIDA*^®^ *QUICK* SCAN and qPCR can be used for screening high DON levels in cereal grain samples, but the samples with DON levels close to the EU limit, should be checked by e.g., GC-MS in order to avoid false positive or false negative results and even then some cases may be difficult.

Only low levels of *F. culmorum* DNA were found in a few oat samples (Tables S1–S5) and no correlation was found between *F. culmorum* DNA and DON levels. *F. graminearum* DNA levels were in all cases in agreement with DON levels, when DON was measured by GC-MS. When compared to *RIDA*^®^ *QUICK* SCAN kit results (DON) the variation in DNA levels was much higher. So, the homogenization of the oats flour by sieving seems to be important to achieve reproducible DON and *F. graminearum* DNA levels. According to Gagkaeva *et al.* [53] filtering of oats flour with a 0.4 mm sieve after milling decreases *Fusarium* biomass connected to hulls, which would mean that flour particles from hulls are bigger than other flour particles. Based on our results *F. graminearum* is clearly the main DON producer in Finnish oats.

There was also a significant correlation (Figure 3) between the combined T-2 and HT-2 and combined *F. langsethiae* and *F. sporotrichioides* DNA levels in 2010–2012. For the combined data of the years 2010–2012 the *R*^2^ value was 0.40 ***. The high DNA levels in oat samples found by TMLAN primers and probe were mainly due to high *F. langsethiae/F. sporotrichioides* contamination in the outer grain layer, which is removed during the de-hulling process before they are used for food [54]. The results (Figure 3) are in agreement with the previous results of Yli-Mattila *et al.* [14,32,38].

### 3.2. Chemotypes of *Fusarium* Species in Finland during the Years 2010–2012

3ADON was found in all grain samples with high DON level, but no 15ADON was found (Tables S1–S4). This is supporting the previous results [13,17,38], according to which the 3ADON molecular chemotype is dominant in northern Europe. NIV was probably produced by *F. poae* (Yli-Mattila *et al.*, [14,55,56]), while *F. langsethiae* is the most common T-2/HT-2 producer of Finnish oats.

## 4. Conclusions

Based on the results of the present and previous works of our group and other researchers, we suggest that there are two main populations of *F. graminearum* in Europe (Figure 1). The population of the 3ADON chemotype is dominant in northern Europe and has recently been spreading from Finland to North-western Russia [6], while the population of 15ADON chemotype is dominant in Central and Southern Europe and has been spreading to Denmark. In northern Europe DON and *F. graminearum* levels are highest in oats (e.g., [13]), while in other parts of Europe *F. graminearum* and DON levels are highest in wheat, suggesting that the northern *F. graminearum* population may be more specialized to oats, while the southern population may be more specialized to wheat. Further work is required to confirm this idea.

We also suggest that the homogenization of the oats flour by milling with a 1 mm sieve seems to be important for the reproducibility of DON and *F. graminearum* DNA levels, which is shown by a higher correlation between DON and *F. graminearum* DNA levels in oats grain samples sieved during milling.

## Supplementary Materials

Supplementary File 1
