# Supplementary material for: Molecular Quantification and Genetic Diversity of Toxigenic Fusarium Species in Northern Europe as Compared to Those in Southern Europe
Source: Microorganisms. 2013 Dec 3;1(1):162–74. doi: 10.3390/microorganisms1010162 (PMC5029496; doi:10.3390/microorganisms1010162)
Supplement: Supplementary File 1 [file microorganisms-01-00162-s001.pdf]

Supplementary Material

Table S1. Oats samples of the year 2010. DNA (ng/μL in Qubit and 10^{-6} ng·ng^{-1} in qPCR) ja and toxins (μg/kg). The same units were also used in Tables S2–S5. The DNA of strains *F. graminearum* strains G-243, *F. sporotrichioides* strain S55 and *F. culmorum* strain were used as standards in qPCR. No DAS, FX and 15ADON was not detected in any sample. n.d. = not detected.

Sample	DNA (Qubit)	Cultivar	Origin	DON	3ADON	NIV	T-2 + HT-2	TMLAN	TMFg12	MGBculm
270-12	5	Fiia	Etelä-Pohjanmaa	82	n.d.	n.d.	0	2900	370	n.d.
252-7	9.5	Roope	Varsinais-Suomi	520	26	430	890	880000	<140	n.d.
252-8	4	Belinda	Varsinais-Suomi	230	<25	n.d.	1000	2000000	0	n.d.
252-10	2.5	Eemeli	Satakunta	150	<25	63	570	730000	<50	<5
252-20	12	Eemeli	Pirkanmaa	52	<25	77	560	1600000	470	0
252-21	12	Leila	Pirkanmaa	140	<25	n.d.	2370	2900000	<50	0
270-5	6.5	Eemeli	Pirkanmaa	250	<25	42	820	1100000	1400	8
252-22	13	Eemeli	Pirkanmaa	180	<25	90	1010	410000	150	0
270-10	15	Belinda	Etelä-Pohjanmaa	140	<25	80	660	650000	400	<5
320-15	9.5	Marika	Etelä-Pohjanmaa	64	<25	43	610	830000	880	0
S55	1.6	-	-	-	-	-	-	-	-	-
G243	0.5	-	-	-	-	-	-	-	-	-
C192	0.5	-	-	-	-	-	-	-	-	-

Table S2. Oats samples of MTT in 2011. No DAS, FX and 15ADON was found in any sample. n.d. = not detected. Samples with high T-2 + HT-2 levels.

Sample	Cultivar	Origin	DON	3ADON	NIV	T-2	HT-2	T-2 + HT-2	TMFg12	TMLAN	MGBculm
320-4	Fiia	Etelä-Savo	310	36	80	110	400	510	-	5083	-
191-4	Ringsaker	Etelä-Pohjanmaa	400	33	32	200	340	540	-	7497	-
280-7	Belinda	Satakunta	34	n.d.	n.d.	100	490	590	-	3023	-
280-10	Ringsaker	Häme	350	33	130	180	420	600	-	3857	-
280-9	Peppi	Häme	<25	n.d.	<25	190	530	720	-	2030	-
280-8	Venla	Satakunta	96	n.d.	n.d.	190	550	740	15	2724	n.d.
320-5	Roope	Keski-Suomi	710	96	41	200	590	790	203	8812	-
302-10	Belinda	Etelä-Pohjanmaa	240	34	100	170	630	800	40	22368	-
343-12	Eemeli	Etelä-Pohjanmaa	1200	270	39	330	590	920	626	13419	35
302-3	Belinda	Kymenlaakso	440	54	<25	200	730	930	148	13981	-
302-6	Peppi	Pohjois-Savo	220	25	n.d.	320	610	930	541	10582	47
343-7	Fiia	Etelä-Savo	110	<25	30	220	760	980	34	30661	-
302-11	SW Vaasa	Keski-Pohjanmaa	87	n.d.	26	330	790	1120	-	6635	-
280-11	Fiia	Pirkanmaa	550	45	<25	480	1100	1580	-	4212	-

Table S3. Oats samples of MTT in 2011. No DAS, FX and 15ADON was found in any sample. n.d. = not detected. Samples with high DON levels.

Sample	Cultivar	Origin	DON	3ADON	NIV	T-2	HT-2	T-2 + HT-2	TMFg12	TMLAN	MGBculm
280-4	Iiris	Varsinais-Suomi	2100	190	33	62	170	232	-	2529	-
302-12	Venla	Kainuu	2200	340	n.d.	n.d.	n.d.	-	-	1189	-
251-3	Iiris	Häme	2300	150	n.d.	57	250	307	467	-	-
233-15	Veli	Etelä-Pohjanmaa	2700	400	75	44	140	184	345	-	n.d.
280-13	Peppi	Pohjois-Savo	3000	280	65	n.d.	n.d.	n.d.	637	-	-
280-18	Fiia	Etelä-Pohjanmaa	3400	330	50	57	130	187	549	-	<10
320-6	Roope	Keski-Suomi	4000	460	160	63	140	203	1031	-	<10
302-5	Peppi	Pohjois-Savo	4900	440	110	68	110	178	1940	-	78
320-2	Veli	Satakunta	8800	740	<25	120	360	480	3155	4372	n.d.
320-7	Iiris	Etelä-Pohjanmaa	13000	1600	30	54	190	244	5180	4832	47

Table S4. Oats samples of MTT in 2012. No DAS, FX and 15ADON was found in any sample. n.d. = not detected. ? = cultivar not known.

Sample	Cultivar	Origin	DON	3ADON	NIV	T-2	HT-2	TMFG12	TMLAN	MGBculm
260-1	Veli	Varsinais-Suomi	28	n.d.	28	n.d.	<25	415	-	n.d.
260-3	Marika	Etelä-Pohjanmaa	1700	260	<25	46	86	9270	-	<50
278-2	Belinda	Satakunta	<25	n.d.	39	270	1100	268	109800	n.d.
278-3	Fiia	Oulu	570	81	n.d.	n.d.	n.d.	2790	-	n.d.
297-1	Eemeli	Uusimaa	n.d.	n.d.	n.d.	340	410	516	35900	n.d.
297-10	Fiia	Etelä-Pohjanmaa	530	63	55	<25	55	1770	-	n.d.
297-4	Peppi	Satakunta	1900	270	<25	n.d.	<25	19400	830	n.d.
297-5	Ivory	Häme	1100	120	n.d.	120	410	87100	17000	n.d.
297-7	Venla	Pohjois-Savo	58	n.d.	n.d.	36	29	406	921	n.d.
297-8	Venla	Pohjois-Karjala	270	59	<25	n.d.	<25	1980	-	n.d.
297-9	Belinda	Keski-Suomi	170	<25	n.d.	1300	2500	1830	-	n.d.
309-1	Belinda	Uusimaa	560	86	57	140	420	4570	36630	n.d.
309-14	Peppi	Pohjois-Savo	2600	490	n.d.	29	51	15000	1701	83
309-15	Venla	Pohjois-Karjala	700	81	n.d.	n.d.	<25	3720	-	n.d.
309-17	Venla	Pohjois-Karjala	88	<25	27	n.d.	n.d.	731	-	n.d.
309-18	Peppi	Keski-Suomi	1400	180	n.d.	<25	34	15800	-	n.d.
309-20	Peppi	Etelä-Pohjanmaa	610	65	n.d.	1100	2000	4980	68900	n.d.
309-21	Fiia	Etelä-Pohjanmaa	2200	350	n.d.	<25	50	8350	883	n.d.
309-22	Fiia	Etelä-Pohjanmaa	130	<25	n.d.	31	70	347	-	n.d.
309-24	Akseli	Oulu	240	25	n.d.	32	63	496	-	n.d.
309-5	Peppi	Varsinais-Suomi	2200	280	n.d.	<25	50	9040	2608	n.d.
309-6	Belinda	Satakunta	760	75	n.d.	27	130	4000	9886	n.d.
309-7	Venla	Satakunta	1700	280	n.d.	<25	29	7100	-	n.d.
309-8	Belinda	Häme	580	86	n.d.	<25	57	2800	-	n.d.
309-9	Belinda	Pirkanmaa	890	98	35	n.d.	n.d.	3400	-	n.d.
353-1	Fiia	Satakunta	110	n.d.	50	43	86	563	-	n.d.
353-18	Fiia	Etelä-Savo	2100	290	<25	76	60	15800	-	n.d.
353-19	Akseli	Etelä-Savo	160	<25	<25	<25	<25	1100	-	n.d.
353-20	Peppi	Pohjois-Savo	6200	930	44	n.d.	<25	80500	-	n.d.

Table S4. *Cont.*

Sample	Cultivar	Origin	DON	3ADON	NIV	T-2	HT-2	TMFG12	TMLAN	MGBculm
353-23	Peppi	Keski-Suomi	630	57	57	120	130	2030	4978	n.d.
353-25	Peppi	Etelä-Pohjanmaa	1400	190	<25	86	100	8090	1910	n.d.
353-26	Marika	Etelä-Pohjanmaa	2200	210	n.d.	<25	37	10100	687	88
353-28	Viviana	Etelä-Pohjanmaa	1700	150	n.d.	67	90	11600	-	n.d.
353-31	Aarre	Keski-Pohjanmaa	1300	120	59	n.d.	<25	5280	-	n.d.
353-33	?	Keski-Pohjanmaa	1800	190	n.d.	n.d.	n.d.	12300	-	n.d.
353-35	Eemeli	Oulu	1400	220	51	n.d.	n.d.	2970	-	n.d.
353-39	Veli	Oulu	1600	200	200	n.d.	<25	6180	101	n.d.
353-4	Fiia	Satakunta	200	35	n.d.	<25	71	835	1464	<50
353-40	Veli	Oulu	550	73	27	38	69	-	610	n.d.
353-9	Peppi	Häme	550	63	n.d.	<25	<25	-	68	n.d.

Table S5. Oats samples of a food company in 2012. DON was analyzed by *RIDA*® *QUICK* SCAN kit. n.d. = not detected.

Sample	Origin	DON	TMFG12	TMLAN	MGBculm
1	Laitila	1000	197	1303	n.d.
2	Parkano	1000	2	1246	n.d.
3	Loimaa	3520	245	1264	n.d.
4	Ulvila	1380	967	2547	n.d.
5	Pöytyä	>5500	647	214	n.d.
6	Somero	>5500	1057	<4	n.d.
7	Urjala	1320	33	1015	n.d.
8	Pirkkala	1800	429	218	71
9	Ypäjä	530	69	1018	n.d.
10	Orivesi	3150	163	150	<50
11	Nilsiä	1820	231	0	n.d.
12	Pori	3430	158	2324	n.d.
13	Akaa	2310	20	1322	n.d.
14	Ilmajoki	4980	641	26000	n.d.
15	Orivesi	710	99	284	n.d.
16	Punkalaidun	1590	147	636	n.d.
17	Forssa	760	63	2848	n.d.
18	Saastamala	5330	774	946	n.d.
19	Nakkila	1590	1038	868	n.d.
20	Hämeenlinna	2310	26	6878	n.d.

© 2013 by the authors; licensee MDPI, Basel, Switzerland. This article is an open access article distributed under the terms and conditions of the Creative Commons Attribution license (http://creativecommons.org/licenses/by/3.0/).
